# On the choice of metric in gradient-based theories of brain function

**DOI:** 10.1371/journal.pcbi.1007640

**Published:** 2020-04-09

**Authors:** Simone Carlo Surace, Jean-Pascal Pfister, Wulfram Gerstner, Johanni Brea

**Affiliations:** 1 Department of Physiology, University of Bern, Bern, Switzerland; 2 Institute of Neuroinformatics and Neuroscience Center Zurich, University Zurich and ETH Zurich, Zurich, Switzerland; 3 School of Computer and Communication Sciences and Brain Mind Institute, School of Life Sciences, École Polytechnique Fédérale de Lausanne, Lausanne, Switzerland; University of Toronto, CANADA

## Abstract

This is a *PLOS Computational Biology* Education paper.

The idea that the brain functions so as to minimize certain costs pervades theoretical neuroscience. Because a cost function by itself does not predict how the brain finds its minima, additional assumptions about the optimization method need to be made to predict the dynamics of physiological quantities. In this context, steepest descent (also called gradient descent) is often suggested as an algorithmic principle of optimization potentially implemented by the brain. In practice, researchers often consider the vector of partial derivatives as the gradient. However, the definition of the gradient and the notion of a steepest direction depend on the choice of a metric. Because the choice of the metric involves a large number of degrees of freedom, the predictive power of models that are based on gradient descent must be called into question, unless there are strong constraints on the choice of the metric. Here, we provide a didactic review of the mathematics of gradient descent, illustrate common pitfalls of using gradient descent as a principle of brain function with examples from the literature, and propose ways forward to constrain the metric.

## Author summary

A good skier may choose to follow the steepest direction to move as quickly as possible from the mountain peak to the base. Steepest descent in an abstract sense is also an appealing idea to describe adaptation and learning in the brain. For example, a scientist may hypothesize that synaptic or neuronal variables change in the direction of steepest descent in an abstract error landscape during learning of a new task or memorization of a new concept. There is, however, a pitfall in this reasoning: a multitude of steepest directions exists for any abstract error landscape because the steepest direction depends on how angles are measured, and it may be unclear how angles should be measured. Many scientists are taught that the steepest direction can be found by computing the vector of partial derivatives. But the vector of partial derivatives is equal to the steepest direction only if the angles in the abstract space are measured in a particular way. In this article, we provide a didactic review of the mathematics of finding steepest directions in abstract spaces, illustrate the pitfalls with examples from the neuroscience literature, and propose guidelines to constrain the way angles are measured in these spaces.

## Introduction

The minimization of costs is a widespread approach in theoretical neuroscience [1–5]. Cost functions that have been postulated range from energy consumption, free energy, negative entropy, and reconstruction error to distances between distributions that form representations of the world [1–3, 5–18]. In some cases, cost as performance of a biological system is measured in comparison to the absolute physical minimum [5] or an information theoretic optimum [1–3] without addressing the question of how a solution at or close to the minimum can be found. In other cases, cost is used to derive algorithms that move the system closer to the minimum [6–20]. In the second case, predictions entail update rules of neuronal quantities, e.g., firing rates of neurons [17, 18], or differential equations for the time evolution of synaptic weights [6–16, 19, 20].

Optimization methods to train neural network models are often taken from machine learning, a field that has had intense interactions with theoretical and computational neuroscience [21, 22]. A successful method in machine learning—despite its simplicity—has been the method of (stochastic) steepest descent or gradient descent [23].

**Fig 1:**
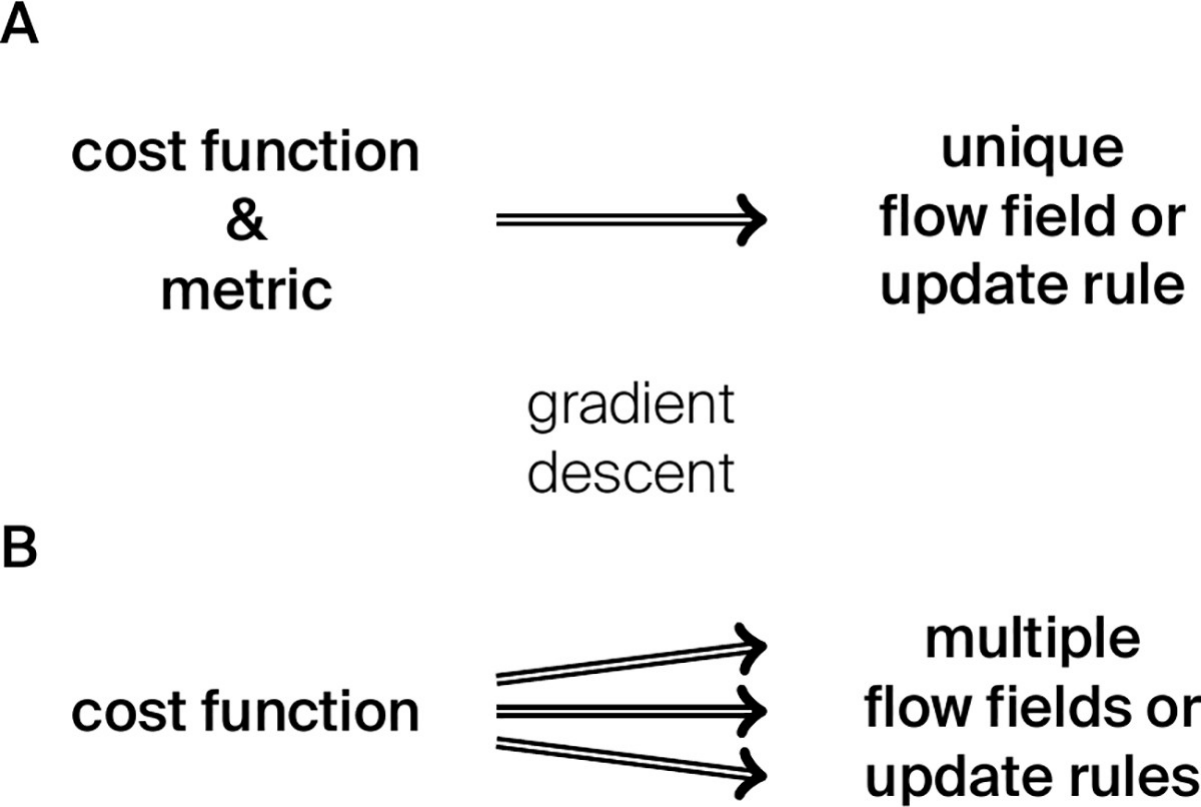
The main message of this text. (A) A cost function and a metric together determine a unique flow field and update rule, given by gradient descent on the cost function in that metric. (B) For a given cost function, there are infinitely many different flow lines and update rules (one for each choice of the metric) that lead to the minima of the cost function by gradient descent.

Gradient descent and steepest descent are the same because the negative gradient points in the direction of steepest descent (see Eq 7). Often the direction of gradient descent is visualized as a vector orthogonal to the contour lines of the cost function. The notion of orthogonality, however, assumes a Riemannian metric (also known as inner product or scalar product in vector spaces). The Riemannian metric enters also in an alternative but equivalent definition of the direction of steepest descent: the direction of steepest descent produces the greatest absolute decrease of the cost function for a step of a fixed (and small) size, in which the step size is determined by the choice of the Riemannian metric. Thus, a cost function by itself does not predict the trajectories that lead to its minima through steepest descent; however, a cost function combined with a metric does (see Fig 1).

**Fig 2:**
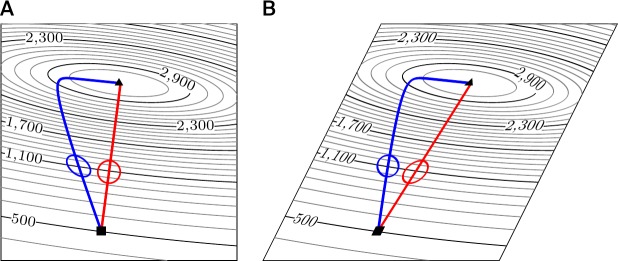
The wicked-map problem. (A) An ambitious mountaineer may follow the gradient in Euclidean metric to reach the mountain top (red route from square to triangle). Because the map is plotted in Cartesian coordinates, the route stands perpendicular to the contour lines. (B) If the ambitious mountaineer does not realize that a map given by a wicked hotelier is sheared, the blue route would be chosen, as it is now the one that stands perpendicular to the contour lines in the sheared map. The blue route corresponds to gradient ascent in another metric. Of course, each route on the normal map could be transformed to the sheared map and vice versa, but what looks like naive (Euclidean) gradient ascent in one map may look different in another map.

Why do we normally not think of the metric as an important and essential quantity? The physical space that surrounds us, at the scales that we encounter in everyday life, is Euclidean. Thus, a mountaineer who would like to determine the direction of steepest ascent of the terrain refers to Euclidean geometry. In this case, the steepest direction is unambiguous because the way to measure distances is intrinsic to the space and not merely an artifact of using a particular set of coordinates. On a map that faithfully represents Euclidean geometry, i.e., preserves angles and lengths up to some scaling factor, the mountaineer may find the steepest direction by drawing a curve that runs perpendicular to the contour lines (see Fig 2A, red route). But if a wicked hotelier gave the mountaineer a map that does not faithfully represent Euclidean geometry, another route would be chosen when planning the route as perpendicular to the contour lines (see Fig 2B, blue route). We will refer to this as the “wicked-map problem” in the following.

What may look obvious in the context of hiking maps can be confusing in contexts in which it is less clear how to draw a sensible map, i.e., how to choose a natural parametrization of an observed phenomenon. We will discuss how naive gradient ascent or descent as taught in text books (e.g., [4, 23]) is susceptible to the wicked-map problem. Although it is simple to display the same path in different maps by following standard transformation rules, the choice of an appropriate metric remains a challenge. In other words, how should one know a priori which metric is most appropriate to predict a route with gradient ascent dynamics? We will illustrate the problems around gradient ascent and descent with three examples from the theoretical neuroscience literature and discuss ways forward to constrain the choice of metric.

### The gradient is not equal to the vector of partial derivatives

Given a cost function *C*(**x**) that depends on variables **x** = (*x*_1_,…,*x*_*N*_), where the variables *x*_*i*_ could be synaptic weights or other plastic physiological quantities, naive gradient descent dynamics is sometimes written as [4, 23]

$$x_{i}\to x_{i}-\tilde{\eta }\frac{\partial C(x)}{\partial x_{i}},$$ (1)

or in continuous time

$$\frac{d}{dt}x_{i}(t)=-\eta \frac{\partial C(x)}{\partial x_{i}},$$ (2)

where $\tilde{\eta }$ and *η* are parameters called learning rate. As we will illustrate in the course of this section, this has two consequences:

- The wicked-map problem: the dynamics in Eq 1 and Eq 2 depend on the choice of the coordinate system.
- The “unit problem”: if *x*_*i*_ has different physical units than *x*_*j*_, the global learning rate *η* should be replaced by individual learning rates *η*_*i*_ that account for the different physical units.

In the section “What is the gradient, then? How to do steepest descent in a generic parameter space,” we will explain the geometric origin of these problems and how they can be solved.

The wicked-map problem often occurs in combination with the unit problem, but it is present even for dimensionless parameters. The parameters or coordinates that are used in a given problem are mostly arbitrary; they are simply labels attached to different points—whereas the points themselves (for example, the position of the mountaineer) have properties independent of the parameters chosen to represent them. For example, it is common to scale the variables or display a figure in logarithmic units or simply display them in a different aspect ratio (transformations like the shearing transformation in Fig 2). The predictions of a theory should be independent of the choice of parametrizations, even if there seems to be a canonical choice of parametrization, as in the case of hiking maps. In the example with the mountaineer, a theory should either predict the blue path or the red path, independently of which map is used. Only a deficient theory predicts the red path if one parametrization is used and the blue path if another parametrization is used. Similarly, if the dynamics of a biological system can be written as gradient descent on a given objective function, nature has chosen one specific metric, and the predictions of our theories should not depend on our choice of the coordinate system. However, as we will show below, a rule, such as Eq 2, that equates the time derivative of a coordinate with the partial derivative of a cost function (times a constant) is not preserved under changes of parametrization (see Fig 2A and 2B).

In order to address the unit problem, we can normalize each variable by dividing by its mean or maximum so as to make it unitless. However, this merely replaces the choice of an arbitrary learning rate *η*_*i*_ for each component by the choice of an arbitrary normalizing constant for each variable.

### Artificial examples

To illustrate the wicked-map problem, let us first consider the minimization of a (dimensionless) quadratic cost *C*(*x*) = (*x*−1)^2^, where *x*>0 is a single dimensionless parameter. The derivative of *C* is given by *C*′ (*x*) = 2*x*−2. Naive gradient descent minimization according to Eq 2 yields $\eta^{-1}\frac{d}{dt}x(t)=-C^{\prime }\;(x(t))=2-2x(t)$ with solution *x*(*t*) = 1+*e*^−2*ηt*^ for initial condition *x*(0) = 2.

Because *x* is larger than zero and dimensionless, one may choose an alternative parametrization $\tilde{x}=\sqrt{x}$. The cost function in the new parametrization reads $\tilde{C}(\tilde{x})=({\tilde{x}}^{2}-{1)}^{2}$, and its derivative is given by ${\tilde{C}}^{\prime }\;(\tilde{x})=4\tilde{x}({\tilde{x}}^{2}-1)$. In this parametrization, it may be argued that a reasonable optimization runs along the trajectory $\eta^{-1}\frac{d}{dt}\tilde{x}(t)=-{\tilde{C}}^{\prime }(\tilde{x}(t))=-4\tilde{x}({\tilde{x}}^{2}-1)$ with solution $\tilde{x}(t)=\frac{1}{\sqrt{-\frac{1}{2}e^{-8\eta t}+1}}$ for initial condition $\tilde{x}(0)=\sqrt{2}$. After transforming this solution back into the original coordinate system with parameter *x*, we see that the original dynamics *x*(*t*) = 1+*e*^−2*ηt*^ and the new dynamics ${\left(\tilde{x}(t)\right)}^{2}=\frac{1}{-\frac{1}{2}e^{-8\eta t}+1}$ are very different. This is expected because the (one-dimensional) vector field −*C*′ (*x*) = 2−2*x* that is used for the first trajectory should behave as $-C^{\prime }\;(x)\to -\frac{\partial \tilde{x}}{\partial x}C^{\prime }\;(x)=\frac{1}{\tilde{x}}-\tilde{x}$ under a change of parametrization, which is different from the vector field $-{\tilde{C}}^{\prime }(\tilde{x})=-4\tilde{x}({\tilde{x}}^{2}-1)$ that is used for the second trajectory. This first, one-dimensional example shows that the naive gradient descent dynamics of Eq 2 does not transform consistently under a change of coordinate system.

**Fig 3:**
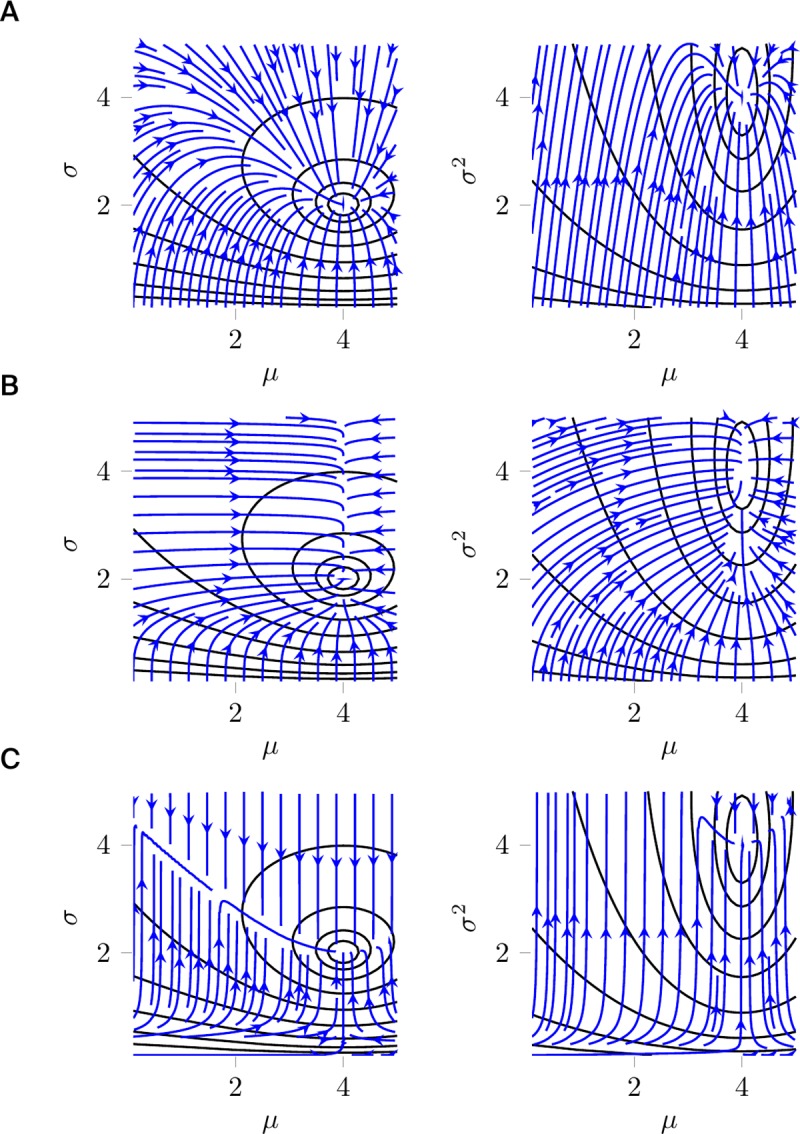
Minimizing the Kullback–Leibler divergence from a fixed normal distribution with mean 4 and standard deviation 2 to a parametrized normal distribution. Equipotential curves in black; flow fields generated by gradient descent in blue with (A) Euclidean metric in mean *μ* and standard deviation *σ*, displayed in *μ*−*σ*–plane (**left**) and *μ*−*σ*^2^–plane (**right**); (B) Euclidean metric in mean *μ* and variance *s* = *σ*^2^, displayed in *μ*−*σ*–plane (**left**) and *μ*−*σ*^2^–plane (**right**); and (C) Euclidean metric in mean *μ* and precision *τ* = 1/*σ*^2^, displayed in *μ*−*σ*–plane (**left**) and *μ*−*σ*^2^–plane (**right**).

As a second example, consider the minimization by gradient descent of the cost function $C(\mu ,\sigma )=D_{\mathrm{KL}}(N(\mu_{0},\sigma_{0})||N(\mu ,\sigma ))$, the Kullback–Leibler (KL) divergence from a fixed normal distribution $N(\mu_{0},\sigma_{0})$ to a normal distribution N(μ,σ) parametrized by its mean *μ* and standard deviation *σ*. A naive gradient descent dynamics would be given by $\frac{d\mu }{dt}=-\frac{\partial C}{\partial \mu }$ and $\frac{d\sigma }{dt}=-\frac{\partial C}{\partial \sigma }$. The corresponding flow field is shown in Fig 3A.

Besides this parametrization, other equivalent ways to parametrize the normal distribution are mean *μ* and variance *s* = *σ*^2^ or mean *μ* and precision *τ* = 1/*σ*^2^. Thus, the function *C* is expressed in the other parametrizations as $\tilde{C}(\mu ,s)=C(\mu ,\sqrt{s})$ or $\bar{C}(\mu ,\tau )=C(\mu ,1/\sqrt{\tau })$. When we apply the same recipe as before to the new parametrizations, we obtain the dynamics $\frac{d\mu }{dt}=-\frac{\partial \tilde{C}}{\partial \mu }$ and $\frac{ds}{dt}=-\frac{\partial \tilde{C}}{\partial s}$ and similar expressions for $\bar{C}$. The corresponding flow fields in Fig 3B and 3C differ from the one obtained with the initial parametrization (Fig 3A) and from each other.

This can also be seen by applying the chain rule to the two sides of $\frac{ds}{dt}=-\frac{\partial \tilde{C}}{\partial s}$ and comparing the result to $\frac{d\sigma }{dt}=-\frac{\partial C}{\partial \sigma }$, the dynamics in the original parametrization. On the left-hand side, we get $\frac{ds}{dt}=\frac{\partial s}{\partial \sigma }\frac{d\sigma }{dt}$, i.e., a prefactor $\frac{\partial s}{\partial \sigma }$. On the right-hand side, we get $-\frac{\partial \tilde{C}}{\partial s}=-\frac{\partial \sigma }{\partial s}\frac{\partial C}{\partial \sigma }$, i.e., a prefactor $\frac{\partial \sigma }{\partial s}$. If the dynamics in the new parametrization would be the same as the one in the initial parametrization, the two prefactors would be the same (see section “Calculations for the artificial example in Fig 3” in S1 Appendix for details).

Despite the different looks of the flow fields resulting from the three different parametrizations, all of them can be seen to describe dynamics that minimize the cost function (Fig 3). However, this example illustrates an important geometrical property that we will come back to later: the differential of a function *f*, i.e., the collection of its partial derivatives, does not transform like a proper vector.

### Gradient descent in neuroscience

In this section we present three examples from published works in which it is postulated that the dynamics of a quantity relevant in neuroscience follows gradient descent on some cost function.

**Fig 4:**
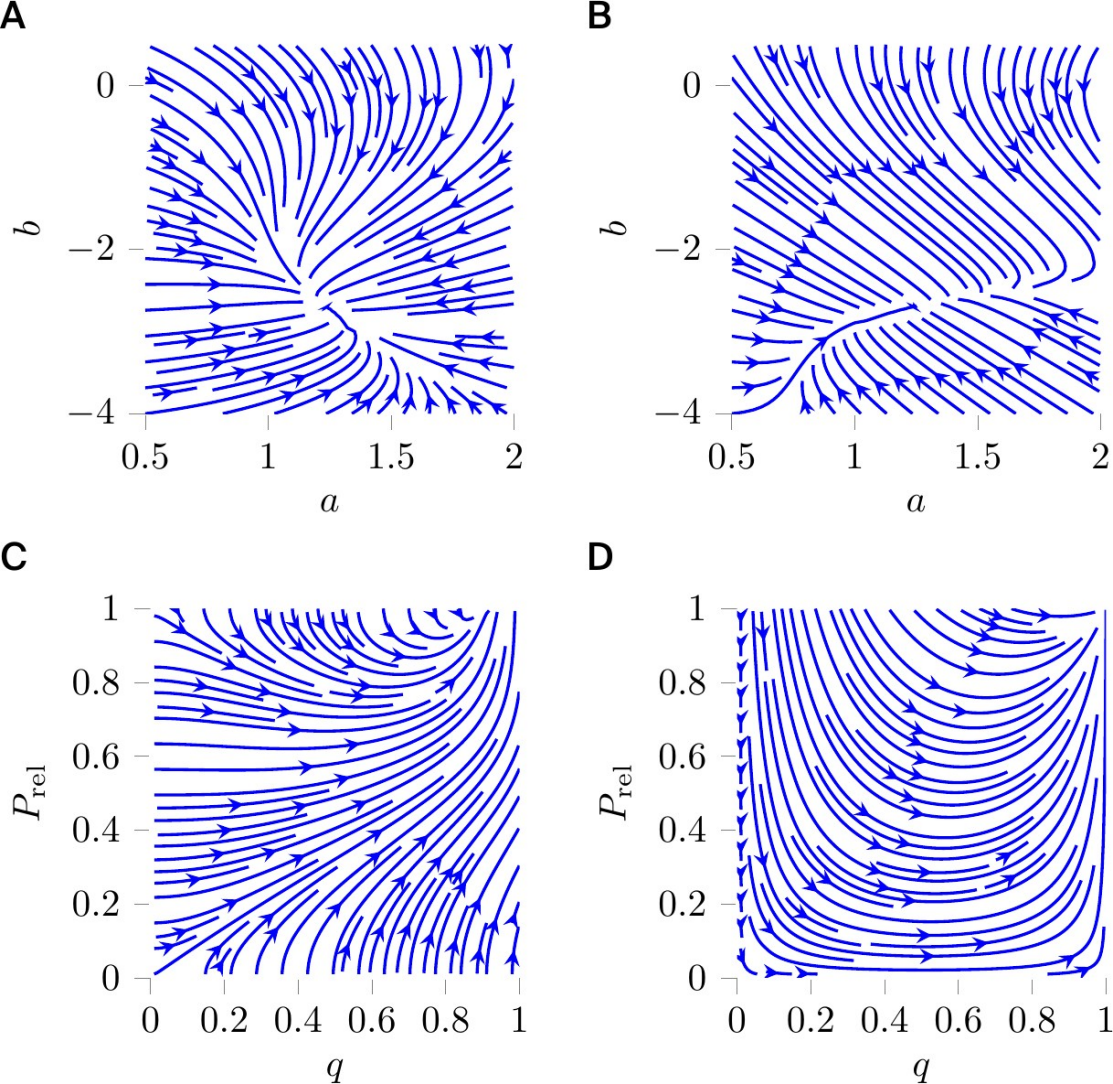
Gradient descent flow fields in neuroscience. (A) Flow of intrinsic plasticity parameters *a* and *b* with Euclidean metric (see Fig 1A in [19]) and (B) with Fisher information metric. (C) Flow of quantal amplitude *q* and release probability *P*_rel_ in a binomial release model of a synapse with Euclidean metric (see Fig 1D in [20]) and (D) with Fisher information metric. For other choices still, see the section “On choosing a metric”.

In 2007, a learning rule for intrinsic neuronal plasticity has been proposed to adjust two parameters *a*,*b* of a neuronal transfer function *g*_*ab*_(*x*) = (1+exp(−(*ax*+*b*)))^−1^ [19]. The rule was derived by taking the derivatives of the KL divergence *D*_KL_(*f*_*y*_‖*f*_exp_) between the output distribution *f*_*y*_, resulting from a given input distribution over *x* and the above transfer function, and an exponential distribution *f*_exp_ with decay parameter *μ*>0. The flow field in Fig 1A of [19] (here Fig 4A) is obtained with the Euclidean metric. If *x* is a current or a voltage, one would encounter the unit problem because *a* and *b* would have different physical units; one may therefore assume that *x* is normalized such that *x*, *a*, and *b* are dimensionless. The wicked-map problem appears because it is unclear whether the Euclidean distance in the (*a*,*b*)-plane is the most natural way to measure distances between the output distributions *f*_*y*_ that are parametrized by *a* and *b*. In fact, in 2013 a different dynamics has been predicted for the same cost function, but under the assumption of the Fisher information metric [24], which can be considered a more natural choice to measure distances between distributions than the Euclidean metric (see Fig 4B). For further details about the Fisher metric, we refer to the section “On choosing a metric”.

Similarly, it has been argued that the quantal amplitude *q* and the release probability *P*_rel_ in a binomial release model of a synapse evolve according to a gradient descent on the KL divergence from an arbitrarily narrow Gaussian distribution with fixed mean *φ* to the Gaussian approximation of the binomial release model [20]. To avoid the unit problem, the quantal amplitude *q* was appropriately normalized. Because *q* and *P*_rel_ parametrize probability distributions, one may also argue for this study that the Fisher information metric (Fig 4D) is a more natural choice, a priori, than the Euclidean metric (Fig 4C), but the corresponding flow fields are just two examples of the infinitely many possible flow fields that would be consistent with gradient descent on the same cost function. Alternatively, one could, e.g., consider metrics that depend on metabolic costs; it may be more costly to move a synapse from release probability *P*_rel_ = 0.9 to release probability *P*_rel_ = 1.0 than from *P*_rel_ = 0.5 to *P*_rel_ = 0.6. If there is no further principle to constrain the choice of metric, data itself may guide the choice of metric (see the section “On choosing a metric”). Surprisingly, the available and appropriately normalized experimental data are consistent with the Euclidean metric in *P*_rel_−*q* space [20], but there are probably not sufficient data to discard a metric based on metabolic cost.

Gradient descent has been popular as an approach to postulate synaptic plasticity rules [7–9, 11–16, 18]. As an example, minimizing by gradient descent the KL divergence from a target distribution of spike trains to a model distribution of spike trains [15] is claimed to lead to a specific plasticity rule with a constant learning rate *η*. This choice of a constant learning rate is equivalent to choosing the Euclidean metric on the weight space. But there is no reason to assume that the learning rate should be constant or the same for each parameter (synaptic weight): one could just as well choose individual learning rates *η*_*ij*_(*w*_*ij*_). This generalization corresponds still to the choice of a diagonal Riemannian metric. But although it is often assumed that the change of a synapse depends only on pre- and postsynaptic quantities (but see [15]), it could be that there is some cross talk between neighboring synapses, which could be captured by nondiagonal Riemannian metrics. This example shows that gradient descent does not lead to unique learning rules. Rather, each postulate of a gradient descent rule should be seen as a family of possibilities: there is a different learning rule for each choice of the Riemannian metric.

### What is the gradient, then? How to do steepest descent in a generic parameter space

In the preceding section, we have shown that the partial derivatives with respect to the parameters do not transform correctly under changes of parametrization (i.e., not as we would expect for the components of a vector or flow field). In order to work with generic spaces that may carry different parametrizations, it is useful to apply methods from differential geometry.

A Riemannian metric on an *N*-dimensional manifold (an intrinsic property of the space) gives rise to an inner product (possibly position dependent) on ${\mathbb{R}}^{N}$ for each choice of parametrization. The matrix representation of the inner product depends on the choice of parametrization. However, the dependence is such that the result of an evaluation of the inner product is independent of the choice of parametrization. When described in this language, the geometry of the trajectories in the space is therefore independent of parameter choices.

To follow the main arguments of this section, it is not necessary to understand the more detailed treatment of gradients on differentiable manifolds presented in the section “Steepest descent on manifolds” in S1 Appendix. But the interested reader is invited to discover there how the terms in Eq 2 are related to tangent vectors and cotangent vectors and how a gradient can be defined on manifolds that are not vector spaces. In the following, we present a simplified treatment in vector spaces with an inner product.

For a function $f:{\mathbb{R}}^{N}\to \mathbb{R}$ and an inner product $\langle \cdot ,\cdot \rangle :{\mathbb{R}}^{N}\times {\mathbb{R}}^{N}\to \mathbb{R}$, a common implicit definition (e.g., [25]) of the gradient (∇*f*)(**x**) of *f* at point **x** is

$$\langle (\nabla f)(x),u\rangle =\underset{\varepsilon \to 0}{\lim}\frac{f(x+\varepsilon u)-f(x)}{\varepsilon }$$ (3)

for all nonzero vectors **u**≠**0**; i.e., the gradient (∇*f*)(**x**) is the vector that is uniquely defined by the property that its product with any vector **u** is equal to the derivative of *f* in direction **u**.

With the Euclidean inner product ${\langle v,w\rangle }_{E}=\sum_{i=1}^{N}v_{i}w_{i}$, it is a simple exercise to see that the components of the gradient are the partial derivatives. However, with any other inner product ${\langle v,w\rangle }_{G(x)}=\sum_{i,j=1}^{N}v_{i}G_{ij}(x)w_{j}$, characterized by the position-dependent symmetric, positive definite matrix *G*(**x**), the gradient is given by

$$(\nabla f)(x)=G^{-1}(x)\left(\begin{array}{c} \frac{\partial f}{\partial x_{1}}\\ \vdots \\ \frac{\partial f}{\partial x_{N}} \end{array}\right),$$ (4)

i.e., the matrix product of the inverse of *G*(**x**) with the vector of partial derivatives. Note that the inverse *G*^−1^(**x**) is also a symmetric, positive definite matrix. The inverse of *G*(**x**) automatically carries the correct physical units and the correct transformation behavior under reparametrizations; i.e., the components of the matrix *G*(**x**) transform as ${\tilde{G}}_{ij}=\sum_{kl}\frac{\partial x_{k}}{\partial {\tilde{x}}_{i}}\frac{\partial x_{l}}{\partial {\tilde{x}}_{j}}G_{kl}$ under a reparametrization from **x** to $\tilde{x}$ such that the dynamics

$$\frac{d}{dt}x(t)=-\eta (\nabla f)(x(t))$$ (5)

is invariant under a change of parametrization. Following standard nomenclature, we call the gradient induced by the Riemannian metric *G* the Riemannian gradient.

The gradient is used in optimization procedures because it points in the direction of steepest ascent. To see this, we define the direction of steepest ascent

$$s(x)≐\underset{\langle u,u\rangle =1}{argmax}\;\underset{\varepsilon \to 0}{\lim}\frac{f(x+\varepsilon u)-f(x)}{\varepsilon }$$ (6)

as the direction **u** in which the change of the function *f* is maximal. Using the definition of the gradient in Eq 3 and determining the maximum, we find

$$\begin{array}{l} s(x)=\underset{\langle u,u\rangle =1}{argmax}\langle (\nabla f)(x),u\rangle \\ \;=\frac{\left(\nabla f)(x\right)}{\left||(\nabla f)(x)|\right|}, \end{array}$$ (7)

where $||\cdot ||=\sqrt{\langle \cdot ,\cdot \rangle }$ denotes the norm induced by the metric 〈⋅,⋅〉.

### On choosing a metric

Given an arbitrary vector field, one may ask whether it is possible to represent it as a steepest descent on some cost function with respect to some metric. It is well-known that gradient dynamical systems have rotation-free vector fields that rule out periodic orbits [26]. Otherwise, when the metric is already known, there is a systematic way to check whether the vector field can be written as a gradient and to construct a suitable cost function. If the metric is unknown, one may have to construct a metric that is tailored to the dynamical system. We refer to section “Which dynamical systems can be regarded as a gradient descent on a cost function?” in S1 Appendix for further details.

Instead of constructing a custom-made metric for the dynamical system, it may be more desirable (from the perspective of finding the most parsimonious description) to choose a metric a priori and then check whether a given dynamical system has the form of a gradient descent with respect to that metric. Such an a priori choice could be guided, e.g., by biophysical principles and therefore becomes an integral part of the theory. For example, a metric could reflect the equivalence of metabolic cost that is incurred in changing individual parameters. Another example is Weber’s law, which implies that parameter changes of the same relative size are equivalent. This would suggest a constant (but not necessarily Euclidean) metric on a logarithmic scale. A third example is the homogeneity across an ensemble: if there are *N* neurons of the same type and functional relevance, we may want to constrain the metrics to those that treat all neurons identically when changing quantities such as neuronal firing thresholds or synaptic weights.

Even if it does not fully determine the metric, a principle that constrains the class of metrics is very useful when trying to fit the metric to the data (for a given cost function). Without any constraints, the specification of a Riemannian metric for an *n*-dimensional parameter space requires the specification of $\frac{1}{2}n(n+1)$ smooth functions, i.e., the components of the matrix *G* in some coordinate system; these components can be constant or position dependent.

If the parameter space describes a smooth family of probability distributions, the Fisher information matrix provides a canonical Riemannian metric on this manifold. The special status of the Fisher–Rao metric in statistics is due to the fact that it is the only metric (up to scaling factors) that has a natural behavior under sufficient statistics (see, e.g., [27], Theorem 2.6 going back to Chentsov, 1972). Natural behavior of a metric (on the set of probability densities) in this context means informally that the sufficient statistics, viewed as transformations of the state space, induce a corresponding transformation of the probability densities that is distance preserving. The various versions of Chentsov’s theorem characterize the Fisher–Rao metric essentially as the only one having this property. The Riemannian gradient with respect to the Fisher–Rao metric is often called the natural gradient, and it has been applied in machine learning [28–36] and neuroscience [24]. Due to Chentsov’s theorem, the Fisher information metric is regarded as a natural choice, but some authors (including Amari in [27]) seem to use the term natural gradient to more broadly refer to a Riemannian gradient with respect to some metric that obeys some invariance principle. Another metric on probability distributions that has recently gained a lot of attention is the optimal transport or Wasserstein metric [37–39]. However, despite the nice mathematical properties of such metrics and their usefulness for machine learning applications, it is not clear why natural selection would favor them. Therefore, the special mathematical status of those metrics does not automatically carry over to biology or, more specifically, neuroscience.

## Conclusions

The idea that biological systems are operating at some kind of optimality is old and is often mathematically formalized as the minimization of a cost function. However, the process by which a minimum is reached cannot be deduced from the cost function alone. The present paper tries to explain that even the simplest dynamical system, steepest descent, depends on the choice of a Riemannian metric (a type of ruler with which step sizes are measured). We aim to draw the attention of the neuroscience community to this fundamental issue and its implications on the widespread use and interpretation of steepest descent in normative or top-down theories of brain function.

First, as we explain in the section “The gradient is not equal to the vector of partial derivatives,” it is important to make the choice of metric an explicit and integral part of the theory. When steepest descent dynamics are being postulated, such as for the dynamics of firing rates or synaptic weights [7–9, 11–18], a Euclidean metric is often chosen implicitly by equating the gradient and the vector of partial derivatives in some arbitrary parametrization. Because the choice is inadvertent, an important part of the explanation of the dynamics is lacking; because the cost function alone does not yield sufficient data to define a notion of steepest descent, a full explanation must include the reason for choosing any specific metric (Euclidean or otherwise).

Second, the Euclidean metric cannot simply be regarded as a default choice (wicked-map problem), especially (but not only) for spaces of parameters that carry different physical units (unit problem). In practice, the unit problem can be treated with a suitable normalization of the measured quantities [19, 20]. The wicked-map problem, however, remains, and it may be a matter of serendipity to select the parametrization in which naive gradient descent (i.e., using an implicit default choice of the Euclidean metric in the parametrization at hand) is consistent with experimental data.

Third, from the biological perspective, the choice of metric is important because it encodes the relative cost of changing different parameters in the pursuit of an optimum and can significantly alter the prediction of the model regarding the trajectories along which optimization occurs (as opposed to just the targets of the optimization). This circumstance opens up difficulties as well as new possibilities; of course, a pure data-driven inference about the optimality principle (cost function and metric) is more difficult and requires more data if the metric is treated as an unknown quantity as opposed to when it is assumed to be Euclidean. On the other hand, as we argue in the section “On choosing a metric,” the additional freedom lets us inform the modeling process by biophysical knowledge about the relative cost of altering physiological quantities. In addition, dynamics that may appear inconsistent with a steepest descent in a Euclidean metric can be consistent with steepest descent dynamics in a different (biophysically meaningful) metric. It will be interesting to uncover the metrics that are chosen by biology, and to uncover the biophysical principles that underlie these choices.

Although the present article has been focused on steepest descent dynamics exclusively, this is only one class of optimization algorithms, and alternative ones may have to be considered for biological systems. However, the choice of metric (or other additional structures) is also relevant for various other methods: for example, in Hamiltonian optimization methods and gradient descent with momentum [40], the metric appears in the kinetic energy term of the Hamiltonian function, which controls the “inertia” of the various directions in the parameter space, and in second-order methods, a metric is usually required (but this can be relaxed somewhat) to define a coordinate-independent notion of Hessian. Thus, the basic point of this article can be generalized to other forms of optimization: in order to be predictive of the dynamics of physiological quantities, normative or top-down principles must include (besides a cost function) additional structure on the parameter space, and this structure often appears in the form of a Riemannian metric.

## Supporting information

S1 Appendix In S1 Appendix, we provide detailed calculations for the artificial example in Fig 3. We give an introduction to steepest descent on manifolds, and we discuss briefly under which conditions a dynamical system can be regarded as a gradient descent on a cost function.
(PDF)
