# Supplementary material for: On the choice of metric in gradient-based theories of brain function
Source: PLoS Comput Biol. 2020 Apr 9;16(4):e1007640. doi: 10.1371/journal.pcbi.1007640 (PMC7144966; doi:10.1371/journal.pcbi.1007640)
Supplement: S1 Appendix — In S1 Appendix, we provide detailed calculations for the artificial example in Fig 3. We give an introduction to steepest descent on manifolds, and we discuss briefly under which conditions a dynamical system can be regarded as a gradient descent on a cost function. (PDF) [file pcbi.1007640.s001.pdf]

Appendix:
On the choice of metric
in gradient–based theories of brain function

Simone Carlo Surace[1], Jean-Pascal Pfister[1], Wulfram Gerstner[2], Johanni Brea[2,*]

1 Department of Physiology, University of Bern, Bern, Switzerland
and Institute of Neuroinformatics and Neuroscience Center Zurich,
University Zurich and ETH Zurich, Zurich, Switzerland

2 School of Computer and Communication Sciences and Brain Mind Institute,
School of Life Sciences, École Polytechnique Fédérale de Lausanne, Lausanne, Switzerland

* johanni.brea@epfl.ch

Calculations for the artificial example in Figure 3

The cost function is given by

$$f(\mu,\sigma) = \frac{(\mu-\mu_0)^2}{2\sigma^2} + \log\frac{\sigma}{\sigma_0} + \frac{\sigma_0^2}{2\sigma^2} - \frac{1}{2}, \tag{S1 1}$$

and its partial derivatives read

$$\frac{\partial f}{\partial \mu} = \frac{\mu-\mu_0}{\sigma^2} \tag{S1 2}$$

$$\frac{\partial f}{\partial \sigma} = -\frac{(\mu-\mu_0)^2 + \sigma_0^2 - \sigma^2}{\sigma^3}. \tag{S1 3}$$

The partial derivatives in alternative parametrizations can be computed either by using the chain rule, e.g. with $s = \sigma^2$

$$\begin{aligned}\frac{\partial \tilde{f}}{\partial s}(\mu,s) &= \frac{\partial \sigma}{\partial s}(s)\frac{\partial f}{\partial \sigma}(\mu,\sqrt{s}) \\ &= -\frac{1}{2\sqrt{s}} \times \frac{(\mu-\mu_0)^2+\sigma_0^2-s}{s^{3/2}} \\ &= -\frac{(\mu-\mu_0)^2+\sigma_0^2-s}{2s^2},\end{aligned} \tag{S1 4}$$

or by expressing the function in terms of the new coordinates, e.g. with $\tau = 1/\sigma^2$

$$\begin{aligned}\bar{f}(\mu,\tau) &= f(\mu, 1/\sqrt{\tau}) \\ &= \frac{1}{2}(\mu-\mu_0)^2\tau - \log\sigma_0\sqrt{\tau} + \frac{\sigma_0^2\tau}{2} - \frac{1}{2}\end{aligned} \tag{S1 5}$$

and then calculating the derivative directly:

$$\frac{\partial \bar{f}(\mu,\tau)}{\partial \tau} = \frac{1}{2}(\mu-\mu_0)^2 + \frac{\sigma_0^2}{2} - \frac{1}{2\tau}. \tag{S1 6}$$

For the partial derivatives with respect to μ we find

$$\frac{\partial \tilde{f}}{\partial \mu} = \frac{\mu - \mu_0}{s} \quad \text{and} \quad \frac{\partial \bar{f}}{\partial \mu} = (\mu - \mu_0)\tau \,. \tag{S1 7}$$

The flows displayed in Figure 3 are obtained by running the following dynamical systems from different initial conditions:

A	B	C
$\frac{d\mu}{dt} = -\frac{\partial f}{\partial \mu}$	$\frac{d\mu}{dt} = -\frac{\partial \tilde{f}}{\partial \mu}$	$\frac{d\mu}{dt} = -\frac{\partial \bar{f}}{\partial \mu}$
$\frac{d\sigma}{dt} = -\frac{\partial f}{\partial \sigma}$	$\frac{ds}{dt} = -\frac{\partial \tilde{f}}{\partial s}$	$\frac{d\tau}{dt} = -\frac{\partial \bar{f}}{\partial \tau}$

Steepest descent on manifolds

Here, we give a short introduction to the *calculus on manifolds* and *differential geometry* that serve as background of this paper. For more details, the reader is referred to the excellent books by Michael Spivak [1] and by Jeffrey M. Lee [2].

In many spaces it is not possible to use the definition of the derivative in direction of $\boldsymbol{u}$ on the right-hand-side of Equation 3 (Main Text). Classical examples of such spaces involve spheres or tori. But also the set $(\mu, \sigma) \in \mathbb{R} \times \mathbb{R}^+$ from our example in Figure 3 together with the canonical vector space operations on $\mathbb{R}$ does not form a vector space, as e.g. multiplication of (μ, σ) with a negative scalar leaves the set of positive standard deviations σ. Nevertheless, these and other spaces are locally similar enough to a linear space to extend ordinary calculus to the calculus on manifolds.

Suppose that we want to define on the manifold M the directional derivative of a function $f : M \to \mathbb{R}$ at point $p \in M$ in the direction $v \in TM_p$, where TM_p is the tangent space in point p. We can then draw a curve γ that runs through p and which has a tangent vector equal to v at that point. For convenience, let $\gamma(0) = p$ and therefore $\left.\frac{d\gamma(t)}{dt}\right|_{t=0} = v$. We then define the differential df_p of f at p as

$$df_p(v) = \frac{d}{dt} f\left(\gamma(t)\right)|_{t=0}, \tag{S1 8}$$

i.e. as a map $df_p : TM_p \to \mathbb{R}$ from the tangent space to the real numbers. It can be shown that this map is linear and well-defined (i.e. it does not depend on the particular choice of γ). In a parametrization $p = \Phi(x) = \Phi(x^1, ..., x^n)$ it reads

$$df_p(v) = \sum_{i=1}^{n} \left.\frac{\partial f}{\partial x^i}\right|_p \left.\frac{dx^i(\gamma(t))}{dt}\right|_{t=0} = \sum_{i=1}^{n} \partial_i f(\Phi(x)) v^i, \tag{S1 9}$$

where $\partial_i = \frac{\partial}{\partial x^i}$ denotes the partial derivative with respect to x^i, dx^i is defined as the linear map from the tangent space to the reals that extracts the i'th component of a vector in tangent space, and v^i is the i'th component of v when expressed in the coordinate basis.

As a linear map from the tangent space to the real numbers, df_p belongs to the cotangent space at p, the dual space T^*M_p of the tangent space TM_p. The cotangent vector df_p is expressed as

$$df_p = \sum_{i=1}^{n} \partial_i f(\Phi(x)) dx^i \tag{S1 10}$$

in local coordinates, with dx^i basis vectors of the cotangent space T^*M_p. We see here that the object we called "vector of partial derivatives" in the main text is actually a cotangent vector.

A tangent vector $v \in TM_p$ can also be seen as a linear map $v : F(M) \to \mathbb{R}$, where $F(M)$ is the space of smooth functions from M to $\mathbb{R}$. In a parametrization this map reads $v(f) = \sum_i v^i \partial_i f(\Phi(x))$. Because of this, it is convenient and common practice to express parametrized vectors in a tangent space as $v = \sum_i v^i \partial_i$ and interpret ∂_i as basis vectors of the tangent space TM_p.

The different geometrical nature of tangent and cotangent vectors is the fundamental reason why a rule such as in Equation 1 (Main Text) or Equation 2 (Main Text) is problematic: on one side of the equation, we have a tangent vector (the velocity vector of the curve along which we want to move), while on the other side we have the differential of the cost function, a cotangent vector. They cannot be equal; they can at most have the same components in *some* coordinates, but this property is lost when changing to a different set of coordinates. Such a rule therefore does not make sense without invoking a preferential choice of coordinates.

Generalization of inner products: Riemannian metrics

In order to obtain a way to transform cotangent vectors into tangent vectors or vice versa and thereby identify them with each other, one needs to define additional structure on the manifold. This structure comes in the shape of what is called a Riemannian metric, which is a map from bivectors (i.e. pairs of tangent vectors) to the real numbers. More specifically, at each point p it specifies a quadratic form or an inner product g_p on the tangent space at that point. In order to qualify for the term *Riemannian*, this quadratic form should in addition be positive definite.[1] Lastly, the metric is usually expected to vary smoothly as a function of the position in the manifold, which means that when it is evaluated on smooth vector fields, the resulting real-valued function is smooth. Given a Riemannian metric g and a point p, a cotangent vector $v^\flat$ is assigned to a tangent vector v in the following way

$$v^\flat : \quad v' \mapsto g_p(v, v'), \tag{S1 11}$$

or in local coordinates

$$v_i^\flat = \sum_{j=1}^n g_{ij} v^j, \quad v^\flat(v') = \sum_{i,j=1}^n g_{ij} v^i v'^j. \tag{S1 12}$$

Since g_p is a bilinear form, we see that both $v^\flat$ itself (as a map from the tangent space to the reals) and the assignment of $v^\flat$ to v are linear maps, and we can also see that the assignment is injective, because if it were otherwise, we could have

$$0 = v_1^\flat(v') - v_2^\flat(v') = g_p(v_1 - v_2, v'). \tag{S1 13}$$

for non-zero $v_1 \neq v_2$ and some non-zero v', which contradicts the positive definiteness of g_p. Since the tangent space and the cotangent space have the same dimension, the assignment is also surjective, and we can therefore define an inverse $^\sharp$ that assigns a tangent vector $\omega^\sharp$ to any cotangent vector ω. An inverse metric g_p^{-1} may then be defined as

$$g^{-1}(\omega_1, \omega_2) = g_p(\omega_1^\sharp, \omega_2^\sharp). \tag{S1 14}$$

In local coordinates, we may write

$$g^{-1}(\omega_1, \omega_2) = \sum_{i,j=1}^n g^{ij} \omega_{1,i} \omega_{2,j},$$

$$\sum_{j=1}^n g_{ij} g^{jk} = \delta_j^k,$$

[1] In many contexts, e.g. in physics, however, metrics are pseudo-Riemannian.

where δ_j^k are the components of the unit matrix (i.e. $\delta_j^k = 1$ if $j = k$ and zero otherwise). Using this inverse metric, $\omega^\sharp$ may be written as

$$\omega^\sharp = g^{-1}(\omega, \cdot), \quad \omega^{\sharp,i} = \sum_{j=1}^{n} g^{ij}\omega_j. \tag{S1 15}$$

Indeed, as a linear map from the cotangent space to the reals, the RHS may be canonically identified with a tangent vector.[2] The isomorphisms $\sharp$ and $\flat$ are known as *musical isomorphisms*, and in terms of local coordinates, they are used to raise and lower indices.

In analogy to the case in $\mathbb{R}^N$ we can now define the gradient on smooth manifolds

$$g_p(\nabla_p f, v) = df_p(v)\,, \tag{S1 16}$$

for all tangent vectors v at point p and, using the definition of the inverse metric, we find

$$\nabla_p f = df_p^\sharp\,. \tag{S1 17}$$

The gradient on a Riemannian manifold

By being given the structure of the Riemannian metric, we obtain a notion of lengths of and angles between tangent vectors, as with any other inner product space. Thus, given a point p, we can ask in which direction the steepest ascent of the function f is. The answer is given by

$$s(p) \doteq \underset{g(v,v)=1}{\operatorname{argmax}}\, df_p(v), \tag{S1 18}$$

where the maximum is taken over all unit-length tangent vectors, and the directional derivative is properly expressed via the action of the differential of f on the tangent vector. This constrained optimization has a cost function

$$\mathcal{L} = df_p(v) - \lambda(g_p(v, v) - 1), \tag{S1 19}$$

where λ is a Lagrange multiplier. In order to solve this optimization problem, we have to compute the differential of $\mathcal{L}$ with respect to v and set it to zero. Because of the linearity of df_p and the symmetry and bilinearity of g_p, we have

$$\begin{aligned}\mathcal{L}(v + v') &= df_p(v + v') \\ &\quad - \lambda(g_p(v + v', v + v') - 1) \\ &= \mathcal{L}(v) + df_p(v') - 2\lambda g_p(v, v') \\ &\quad - \lambda g_p(v', v').\end{aligned} \tag{S1 20}$$

The critical tangent vector v is therefore characterized by the vanishing of the term that is linear in v'

$$d\mathcal{L}(v') = df_p(v') - 2\lambda g(v, v') = 0, \tag{S1 21}$$

to be satisfied by all tangent vectors v'.[3] As we developed above, this equation has a unique solution, given by $v = \frac{1}{2\lambda} df_p^\sharp$, which, when normalized, reads

$$v = \frac{df_p^\sharp}{\sqrt{g_p(df_p^\sharp, df_p^\sharp)}}\,, \tag{S1 22}$$

which points in the same direction as the gradient in Equation S1 17 and generalizes Equation 7 (Main Text) to differential manifolds.

---

[2] For a finite-dimensional smooth manifold, the map $v \mapsto (\omega \mapsto \omega(v))$ is an isomorphism between the tangent space and its double dual.

[3] Note that the tangent space of the tangent space is the tangent space itself.

Which dynamical systems can be regarded as a gradient descent on a cost function?

In some cases we may start with a given dynamical system in the form of a vector field V on some manifold M. The question arises whether we can find a function f and a metric g such that the dynamical system takes the form of a (negative) gradient flow, i.e. $V = -\nabla_g f$. For this question to make sense, we fix an asymptotically stable set S, with domain of attraction A. In other words, we assume that the dynamics converges to a fixed point x_0, when starting from an initial condition $x \in A(x_0) \subset A$.

If g is given but we do not know f, we can compute the one-form $V^\flat$ that is dual to V with respect to g, and check whether it is closed, i.e. whether $dV^\flat = 0$.[4] If $V^\flat$ is indeed closed and the domain of attraction A is contractible (this is always true if S consists of a single point), this implies the existence of a function f, unique up to an additive constant, such that $V^\flat = -df$, and hence $V = -\nabla_g f$, on A. A suitable potential function f may be found by picking a reference point $p_0 \in S$ and integrating $V^\flat$ along any curve that joins p_0 and p. Note that if we change to a different metric g', the corresponding $V^\flat$ might no longer be closed and hence such a potential may cease to exist.

If neither f nor g are given, a sufficient condition for their existence on a compact manifold was given by [3, 4] (see also [5] for a survey): if the vector field is gradient-like[5], then the construction in Theorem B of [3] gives a suitable cost function f. By the remark after Theorem B, a suitable Riemannian metric g exists. However, these conditions are not necessary, since there are gradient vector fields that do not meet the transversality conditions to be gradient-like.[6] However, they are necessary for a gradient vector field that is structurally stable, which is often desirable in practise. On a non-compact manifold it is not known whether we can find a complete metric, but we can always use the construction above on a compact subset. Alternatively one may find a smooth Lyapunov function (this is always possible; see Theorem 3.2 in [6]) and use the method in the next paragraph to construct a suitable metric.

Suppose that a candidate cost function f is given, and a suitable Riemannian metric g is sought. This case is discussed in [7]. A necessary condition for the existence of g is that f is a smooth local Lyapunov function for V, i.e. $f > 0$ and $Vf = df(V) < 0$ on $A \backslash S$, and $f = 0$ and $df = 0$ on S. Away from singular points, i.e. on $A \backslash S$, this is sufficient. One may consider the level sets $f^{-1}(q)$ for $q \in (0, a) = f(A \backslash S)$, which are submanifolds of dimension $n-1$. We may then choose a Riemannian metric on each level set such that it depends smoothly on q, and by declaring V to be orthogonal to the level sets and to have a squared Riemannian length equal to $|df(V)|$. But as the following example shows, this may not work at singular points: suppose that we have the dynamical system $dx/dt = V(x) = -x$ and the function $f(x) = x^4$ on $\mathbb{R}$. This dynamical system has a global attractor at $x = 0$, and f is a global Lyapunov function since we have $f(0) = 0$ as well as $f(x) > 0$ and $df(V) = 4x^3(-x) = -4x^4 < 0$ for all $x \neq 0$. But if we want to write $dx/dt = -df(x)/g(x)$, we obtain $g(x) = -df(x)/V(x) = 4x^2$, which is not a Riemannian metric on $\mathbb{R}$ because it is degenerate at $x = 0$. This shows that in order to obtain a metric on the entirety of A, V and f have to satisfy additional compatibility conditions at singular/critical points, which are discussed in [7].

[4] In three-dimensional space, this reduces to checking whether curl $V = 0$.

[5] This property is expressed in terms of transversality conditions on the vector field, where transversality is the property of two submanifolds to intersect in a way such that their tangent spaces together span the ambient tangent space. A vector field is called gradient-like if it is transversal to the zero section at each fixed point, transversal to the boundary, and the stable and unstable manifolds of each singular point meet transversally.

[6] This is a point of confusion in the nomenclature. It turns out that not even gradient vector fields of Morse functions are gradient-like. The standard counterexample is the gradient field of the height function on an upright 2-torus. However, by Theorem A of [3], a gradient vector field of a Morse function which is transversal to the boundary can be approximated by a gradient-like vector field (which is by definition transverse to the boundary but also has transversal stable and unstable manifolds, see footnote 5).

References

[1] Spivak M. Calculus on manifolds. 5th ed. A modern approach to classical theorems of advanced calculus. Westview Press; 1971.

[2] Lee JM. Manifolds and differential geometry. vol. 107. American Mathematical Soc.; 2009.

[3] Smale S. On gradient dynamical systems. Annals of Mathematics Second Series. 1961;74(1):199–206.

[4] Wall CTC. A Remark on Gradient Dynamical Systems. Bulletin of the London Mathematical Society. 1972;4(2):165–166.

[5] Grines V, Pochinka O. Energy functions for dynamical systems. Regular and Chaotic Dynamics. 2010;15(2):185–193. doi:10.1134/S1560354710020073.

[6] Wilson Jr FW. Smoothing derivatives of functions and applications. Transactions of the American Mathematical Society. 1969;139:413–428.

[7] Wall CTC. Reflections on gradient vector fields. In: Proceedings of Liverpool Singularities Symposium II. Berlin, Heidelberg: Springer, Berlin, Heidelberg; 1971. p. 191–195.
